# Effect of various carbon plate running shoes on peak tibial accelerations, lateral asymmetry and residual shock in running

**DOI:** 10.3389/fspor.2026.1908480

**Published:** 2026-09-04

**Authors:** Marlene Riedl, René Schwesig, Olaf Ueberschär

**Affiliations:** 1 Department of Orthopaedic and Trauma Surgery, Martin Luther University Halle-Wittenberg, Halle, Germany; 2 Department of Engineering and Industrial Design, Magdeburg-Stendal University of Applied Sciences, Magdeburg, Germany; 3 Directorate, Institute for Applied Training Science, Leipzig, Germany

**Keywords:** advanced footwear technology, asymmetry index, biomechanics, inertial measure unit, running gait

## Abstract

Carbon plate running shoes (CPRS) have significantly advanced running shoe technology, but their orthopaedic impact remains controversial. Remarkably, most running-related overuse injuries affect one leg only, although running is generally considered a symmetrical physical activity. Therefore, this study examined whether CPRS affect biomechanical loading and between-leg symmetry. Twenty-two trained amateur triathletes (13 men and 9 women) participated in a two-day trial. On day 1, ventilatory thresholds (VT1 and VT2) were determined through incremental treadmill testing. On day 2, participants completed five 3-min running trials at three different speeds [90% *v*_VT1_, 0.5(*v*_VT1_ + *v*_VT2_), and 100% *v*_VT2_] while wearing four different CPRS models, and their own non-CPRS. Inertial measurement units recorded time-continuous tri-axial tibial accelerations. Results show that peak tibial accelerations and, in parts, also residual shock varied across CPRS models (*p* < 0.021), but lateral asymmetry remained unchanged (*p* > 0.361). Interestingly, the findings varied between sexes and exhibited substantial inter-individual effects. These results suggest that increased injury rates possibly associated with CPRS might not be due to altered between-leg asymmetry, but perhaps because of other factors involving residual shock and/or tibial loading patterns during stance phase. Future research should explore these effects across a broader range of CPRS models.

## 1 Introduction

Since its introduction in 2017 (1, 2), Advanced Footwear Technology (AFT) has become one of the most impactful improvements in running equipment technology, alongside running watches (3, 4). Several new world records have been set, and many amateur runners have started using AFT in their races and training. While many studies have shown that AFT improves running economy (2, 5–7), their biomechanical effects seem to be double-edged, and the underlying biomechanical effects and their orthopaedic implications are a subject of ongoing scientific debate (4, 8, 9).

The prevalence of running-induced overuse injuries, particularly to the Achilles tendon, has been reported to be increasing (10). However, most injury reports related to AFT continue to be constituted by case reports (11). Such injuries generally arise from the repetitive loading of the musculoskeletal system with each stride while there is a mismatch of loading and load-bearing capacity of the involved tissues (12). Despite the high level of cushioning provided by AFT, the biomechanical loads on the lower extremity are not necessarily reduced. In fact, studies have shown that highly cushioned shoes may in fact lead to an increase in impact peaks of ground reaction force (GRF) and loading rates (12). This contradicts the commonly held belief that higher cushioning should result in lower impact forces. Recent research shows that a high midsole stack height may alter peak tibial accelerations (PTAs) (13). In this light, the present study aims to investigate the extent to which different AFT models modify the amplitude of PTA, which form a well-established surrogate measure for external loading and are related to GRF metrics (14–18). It should be noted that PTAs per se may not adequately represent internal bone forces because of substantial internal muscle forces, but they are capable of providing a valid quantitative picture of external dynamics in running, and may potentially be used to elucidate internal loading through specific machine learning approaches (19, 20). Given the focus of this study on the use of carbon plate running shoes (CPRS), this term is employed in the following with preference to the more general AFT for the sake of clarity.

From an orthopaedic perspective, it should be noted that running-induced overuse injuries typically affect only one of the two legs, despite the fact that running is generally considered to be a per se symmetrical sport. It may thus be hypothesised that musculoskeletal or habitual asymmetries have a role to play in the aetiopathogenesis of such injuries. In general terms, asymmetry defined as a functional or morphological difference between the two sides of the body, or two limbs in particular (21). Specifically to bipedal running, kinematic and related kinetic differences between the two legs may manifest themselves as lateral asymmetries, which can be detected by a disparity between the PTAs of the left and the right leg for each step (18, 22). Researchers have examined alterations in this naturally occurring lateral asymmetry in terms of PTA across various surfaces, including concrete, grass, and tartan (18), as well as distinct training sessions (23). Additionally, investigations have been conducted on athletes following injury to ascertain the underlying causes of such occurrences (24). However, the question how kinematic asymmetries, as measurable through PTA, may be altered by CPRS has not been elucidated so far. Therefore, the present study aims to bridge this research gap by investigating how CPRS interact with existing lateral asymmetries in running. Theoretically, both amplification or mitigation, or even their initial introduction would be conceivable effects.

Moreover, this study investigates residual shock as the third biomechanical parameter accessible through peak accelerations. Residual shock is defined as the proportion of tibial acceleration shock that is transmitted to the pelvis, and its quantification has the potential to provide valuable insights into musculoskeletal factors such as limb stiffness, core stability, neuromuscular status of fatigue, or biomechanical running efficiency. Therefore, residual shock has been discussed in the literature as a potentially contributing factor to sustaining running-induced overuse injuries (18, 25, 26). In view of the possible effects of CPRS on PTA, we hypothesise that residual shock may be subject to an alteration by CPRS.

Altogether, this study investigates how CPRS affect PTA amplitudes, their lateral asymmetries and the residual shock at the pelvis, and discusses the findings in the framework of possible biomechanical rationales.

## 2 Methods

### 2.1 Subjects

Following the statistical power analysis put forward by Xiang et al. (2022) for a related research question (13), we set the expected effect size to 0.25 and the statistical power to 0.8. With *α* = 0.05 and *β* = 0.2, a minimum of 21 participants was required for this study. To this end, we recruited 22 amateur runners and triathletes, of whom 13 were males (35.0 ± 8.0 years, 71.9 ± 8.6 kg, BMI 22.7 ± 2.0 kg m^−2^, $\dot{V}O_{2_{\mathrm{peak}}}$ 61.6 ± 5.1 ml kg^−1^ min^−1^) and nine females (29.2 ± 10.3 years, 61.3 ± 5.7 kg, BMI 21.5 ± 1.3 kg m^−2^, $\dot{V}O_{2_{\mathrm{peak}}}$ 52.7 ± 3.1 mL kg^−1^ min^−1^). All athletes had a personal best over 10 km road running of better than 45 min (males, actual sub-cohort mean of 37:00 ± 2:54 min) or better than 50 min (females, 44:06 ± 3:42 min), respectively, and reported an individual average weekly running mileage of at least 20 km (33.3 ± 12.0 km). All runners declared to have been free of injuries for at least three months prior to their participation in the study, and confirmed to be in good health at the days of testing. The study was conducted in accordance with the most recent revision of the Declaration of Helsinki (27), and was approved by the Ethics Committee of the Magdeburg-Stendal University of Applied Sciences (EKIWID-2023-09-001RM). All subjects provided written informed consent prior to their participation.

### 2.2 Footwear conditions

**Figure 1:**
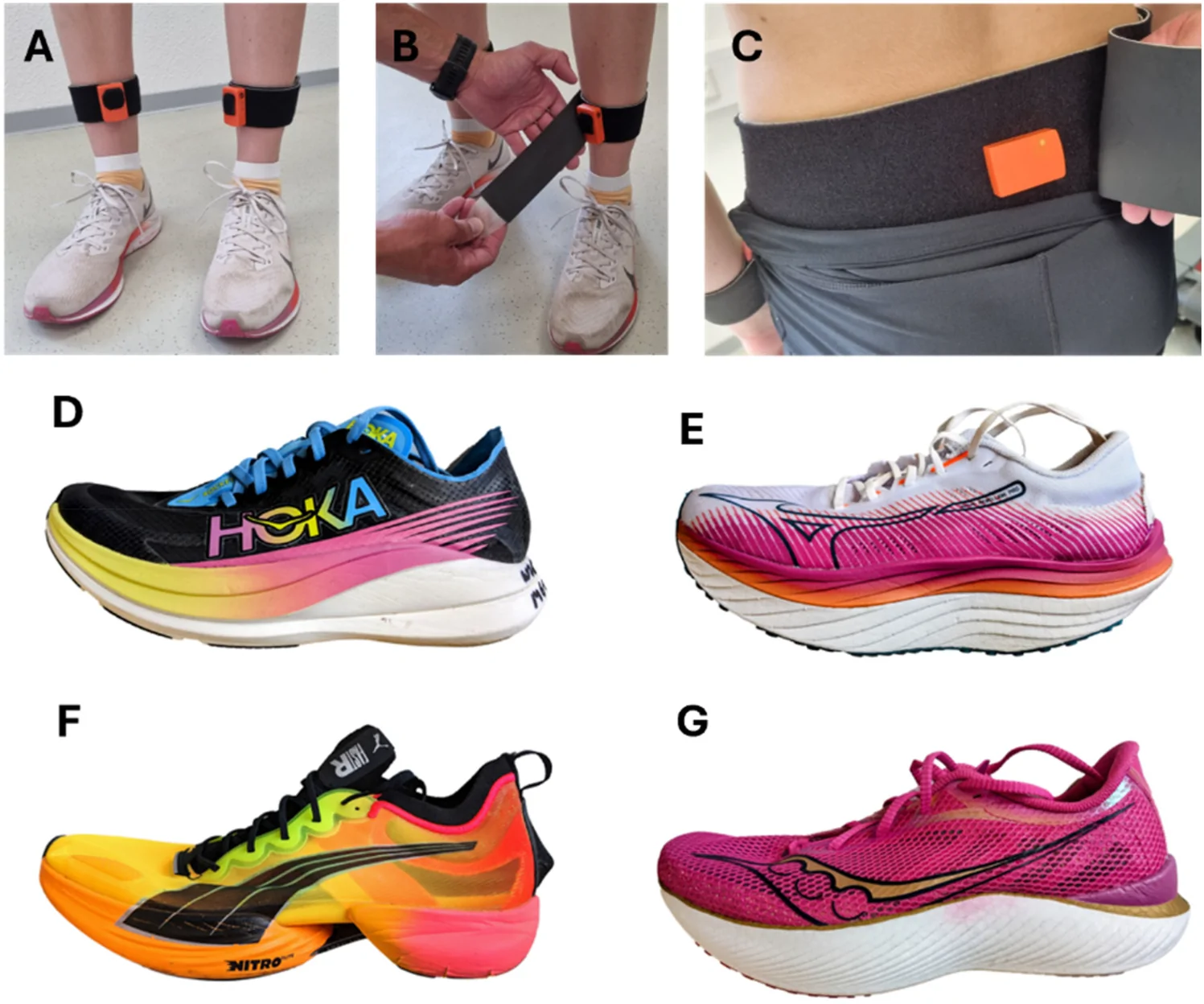
Sensor placement and CPRS footwear conditions. **(A-C)** Placement of the three IMUs at the distal tibiae, and lumbo-sacral region with tightly fitted Velcro straps. **(D–G)** The four used CPRS in this study. Reprinted from Riedl et al. (7), licensed under CC-BY. **(D)** Hoka Rocket X2 (HOK), **(E)** Mizuno Wave Rebellion Pro (MIZ), **(F)** Puma Fast-R Nitro Elite (PUM), and **(G)** Saucony Endorphin Pro 3 (SAU).

Four CPRS from four different manufacturers were included in this study (Figures 1d–g): Hoka Rocket X2 (HOK), Mizuno Wave Rebellion Pro (MIZ), Puma Fast-R Nitro Elite (PUM), and Saucony Endorphin Pro 3 (SAU) (7). Basic structural properties are summarised in Table 1 for the average size of the cohort's males and females. Shoe sizes of the participants ranged from US 7 to US 11.5 for the men, and from US 7 to US 10.5 for the women. The participant's individual own pair of training shoes (OWN) served as an individual reference to the specific asymmetry inherent to each athlete at a given running speed (23). The ranges of basic structural properties for OWN are given in Table 1, with more detailed information on each shoe worn by participants provided in the Supplementary Table 1. Despite the variety in individual OWN models, they essentially meet the recommendations on neutrality and wide availability of simple footwear technology for control study purposes as proposed by Burns et al. (4). More generally, switching from OWN to a current CPRS model pictures a realistic scenario for amateur athletes when changing from non-carbon plate to carbon plate running shoes for competitions.

**Table 1:** Basic structural properties of the four CPRS and the OWN models used.

Property	HOK	MIZ	PUM	SAU	OWN
	Hoka Rocket X2	Mizuno Wave Rebellion Pro	Puma Fast-R Nitro Elite	Saucony Endorphin Pro 3	
Release year^1^	2023	2023	2022	2022	2019–2022
Mass (men US 9.5)*	219 g	218 g	231 g	210 g	220–335 g
Mass (women US 8.5)*	200 g	192 g	202 g	186 g	188–266 g
Heel drop^1^	5.0 mm	4.5 mm	7.5 mm	8.0 mm	4.0–10.0 mm
Midsole foam^1^	PEBA foam	‘Mizuno Enerzy Lite/Lite+’ the latter being PEBA foam	forefoot PEBA foam, heel EVA foam	‘PWRRUNPB’ PEBA foam	
Carbon plate^1^	Carbon plate between two layers of foam	Carbon-infused nylon plate (70% nylon, 30% carbon)	Carbon plate named ‘PWRPLATE’	S-shaped carbon plate	
Upper material^1^	Synthetic mesh	Knitted polyester from elastic thread	Transparent monomesh	Thin, breathable mesh	

* measured in our laboratory; ^1^ data taken from Ref. (28) for HOK, from Ref (29, 30). for MIZ, from Ref (31). for PUM, and from Ref (32). for SAU. The masses shown for OWN represent the average masses as weighed for the cohort of test subjects.

### 2.3 Inertial measurement units (IMUs)

Inertial measurement units (IMUs) of the type Xsens MTw Awinda (Movella Technologies B.V., Enschede, The Netherlands), which have been extensively validated and used in previous research in sports science and biomechanics (18, 24, 33–37) were used. These IMUs permit the non-invasive measurements of time-continuous tri-axial accelerations, angular velocity and magnetic field strength. Their small dimensions (4.7 cm × 3.0 cm × 1.3 cm) and minimal weight (16 g) ensure that they do not distract or interfere with the athletes during the tests. The internal sampling rate of 1000 Hz was downsampled through strapdown integration and sensor fusion to an effective, time-continuous output data rate of 120 Hz, following previous studies (18, 23, 33). Noteworthily, the manufacturer's sensor fusion algorithms preserve actual peak amplitudes and positions despite reduced temporal resolution (18, 38, 39). The axial measurement range is ±16 *g* with 1 *g* ≈ 9.81 m s^−2^, which has been widely shown to suffice for the intended study design (15, 17, 18). Gravity was not removed to ensure that the total acting acceleration was analysed, including both the (dominant) inertial and the gravitational contribution. In this study three IMUs of the described type were fixed noninvasively to three different body segments using tightly fitting Velcro^TM^ straps: Two sensors were positioned on the distal anteromedial sections of the right and left tibia (with the lower edge of the IMU housing being placed 7 cm above the top of the medial malleolus, Figures 1a,b) and on the lumbo-sacral region near L5/S1 (Figure 1c), in line with the placement in previous research (18, 23). By applying these sensor positions, relative soft tissue motion between the IMUs and the bones is minimised (17, 18).

### 2.4 Study design

This study was conducted applying a two-day trial design. On day 1, the initial assessment, the subjects completed a standardised incremental test protocol on a motorised treadmill (Star Trac FreeRunner 10TRx, Core Health 6 Fitness, Vancouver, BC, Canada), set at 1% incline (40), until voluntary exhaustion. In this incremental test, the participants wore their own preferred pair of (non-carbon plate) running shoes with which they would self-reportedly run a race if no CPRS were available. Starting at 6 km h^−1^, the speed was increased in steps of 2 km h^−1^ every 3 min. The first two stages (i.e., at speeds of 6 and 8 km h^−1^, respectively) were used as a standardised warm-up. During the entire test, the heart rate was monitored using a chest strap monitor (Polar H10; Polar Electro Oy, Kempele, Finland), and continuous breath-by-breath cardiopulmonary gas exchange data were collected (MetaMax 3B, CORTEX Biophysik GmbH, Leipzig, Germany).

Based on the results of these tests, the individual running speeds at ventilatory thresholds 1 (*v*_VT1_) and 2 (*v*_VT2_) were derived. If a ventilatory threshold was found within a stage, threshold speed was corrected by linear interpolation between the running speeds of the adjacent stages. Using the experimental values for *v*_VT1_ and *v*_VT2_, three individual running speeds (*v*_1_, *v*_2_ and *v*_3_) were calculated for the second test on day 2:

$$v_{1}\;=\;90\text{\%}\;v_{\mathrm{VT}1},\;v_{2}\;=\;0.5(v_{\mathrm{VT}1}\;+\;v_{\mathrm{VT}2})\;and\;v_{3}\;=\;100\text{\%}\;v_{\mathrm{VT}2}$$ (1)

These speeds were chosen to cover the full spectrum of running speeds that runners typically use in their training and racing, ranging from easy to moderate zone 2 training, via intense zone 3 training, to transition zone 3/ zone 4 racing speed near maximum lactate steady state (41).

On day 2, which took place at least 48 hours after the first day, the same experimental setup was utilised as described for the initial incremental test. This time, however, the athletes performed 5 sessions of 3 × 3 min of treadmill running each, wearing one out of five different pair of shoes in each session in a randomised order (4 CPRS, OWN). The randomisation process was conducted utilising Office 365 Excel (Microsoft Corporation, WA, USA) through the formula input

=SORTBY(A1:A5;RANDARRAY(5)) (2)

employed for each participant, where the cells A1–A5 contain the codes for HOK, MIZ, PUM, SAU, and OWN.

The sessions were run at increasing speeds *v*_1_, *v*_2_ and *v*_3_ in fixed order, resulting in 9 consecutive minutes of running. After each session, a five-minute break was allotted for the athletes to change footwear and achieve a physiological state as close to baseline as possible given the trade-off between practicability and comparability of results. The fixed order of the speeds was chosen so that there was an increasing effort for each specific shoe, and because running at *v*_1_ provided an additional means of active recovery after a previous intense *v*_3_ run. Despite expected to be small, a residual effect of a previous *v*_3_ run on the subsequent *v*_1_ run could not be fully ruled out. Notwithstanding, the randomisation of shoe order among subjects is believed to minimise any relatable effect in this regard.

### 2.5 Data analysis

The initial processing of the raw acceleration data was conducted using a proprietary software (MT Software Suite MTw^TM^ AwindaTM 4.8, Xsens Technologies B.V., Enschede, Netherlands). This software converted the binary data into text format. Subsequent data processing and analysis were performed using a custom software program employing local peak detection in time-continuous acceleration data with self-adjusting thresholds and noise filtering [written in LabVIEW 2023 Q3 (National Instruments, Austin, TX, USA)]. Specifically, the vector magnitudes of triaxial tibial accelerations were calculated using the standard Euclidean norm, i.e.

$$|\vec{a}(t)|=\sqrt{a_{x}{\left(t\right)}^{2}+a_{y}{\left(t\right)}^{2}+a_{z}{\left(t\right)}^{2}},$$ (3)

where $a_{x}$, $a_{y}$ and $a_{z}$ denote the Cartesian vector components in the local reference frame of the IMU sensor. Based on these magnitude time series, a total of 2.5 min of data was extracted from each three-minute segment, with the initial and final 15 seconds excluded from the analysis (i.e., seconds 15–165) to account for any potential accommodation or cessation effects. Using the built-in peak detection procedure of LabVIEW [Peak Detector.VI (42)], peaks were identified in the time series by fitting quadratic polynomials to three sequential data points of the unfiltered raw data (33, 43). The threshold of minimal peak amplitudes was set to 4 *g*, reflecting the observation in our study that at speeds at or beyond *v*_1_, each athlete's impact peaks of each step exceeded at that threshold. The minimum temporal distance between two neighbouring peaks was to set to 50% of the median detected stride duration, thereby minimising the risk of falsely detecting double peaks. In addition, the results for each segment automatically analysed were checked visually, and any incorrectly identified single steps were excluded manually. The median values of the detected peak tibial accelerations (PTA) for the left leg (*a*_left_) and right leg (*a*_right_) were then used to calculate the lateral asymmetry index LTA_3D_) as defined by

$${\mathrm{LTA}}_{3D}:=\frac{a_{\mathrm{left}}-a_{\mathrm{right}}}{\frac{1}{2}(a_{\mathrm{left}}+a_{\mathrm{right}})}.$$ (4)

Notably, median values were used instead of arithmetic means to minimise any potential bias by outliers, while the computation on leg-wise medians was given preference to a step-wise evaluation of Equation (4) to further improve the signal-to-noise ratio. On the level of individual athletes, signed values of LTA_3D_, as defined by Equation (4), were employed for any further exploratory analyses, meaning that both positive (or zero, i.e., non-negative) and negative values may occur.

On the cohort level, however, absolute values of asymmetry indexes aLTA_3D_ defined by

$$\mathrm{aLT}A_{3D}:=|\mathrm{LT}A_{3D}|\geq 0$$ (5)

were used for subsequent statistical analyses to avoid numerical cancelling effects of positive and negative values.

In analogy to PTA, peak sacral accelerations (PSA) were detected for each step based on the time series of triaxial acceleration magnitudes obtained from the IMU worn at the sacrum, and their median values were used for further statistical analysis. Residual shock (*RSh*) in the time domain, reflecting the transmission of the impact shock wave from the tibiae to the pelvis (18), was then calculated using the ratio of median PSA and median PTA amplitudes (44–47), i.e.

$$RSh:=\frac{\mathrm{PSA}}{\mathrm{PTA}},$$ (6)

with PTA denoting the mean of left and right PTA, i.e., $\mathrm{PTA}:=\frac{1}{2}(a_{\mathrm{left}}+a_{\mathrm{right}})$.

In order to determine the dominant leg, the athletes were asked to report their habitually preferred jumping and stance leg if applicable. For further statistical analysis, the product *P* of LTA_3D_ and the leg dominance index *κ* was calculated separately for jumping and stance leg as the product

$$P:={\mathrm{LTA}}_{3D}\cdot \kappa ,$$ (7)

where κ was defined as

$$\kappa :=\left\{ \begin{array}{cc} +1 & \mathrm{if}\;\mathrm{left}\;\mathrm{leg}\;\mathrm{is}\;\mathrm{dominant}\\ \text{-}1 & \mathrm{if}\;\mathrm{right}\;\mathrm{leg}\;\mathrm{is}\;\mathrm{dominant}\\ 0 & \mathrm{otherwise}\;(i.e.,\;\mathrm{indifferent}\;\mathrm{leg}\;\mathrm{dominance}) \end{array}\right..$$ (8)

The rationale behind this definition is as follows: If leg dominance and lateral asymmetry in terms of PTA were stochastically independent, *P* and LTA_3D_ should exhibit the same statistical distribution because no significant shifts in the distribution of positive and negative signs should occur between LTA_3D_ and *P*. That is, runners with a left-dominant lateral asymmetry (i.e., LTA_3D_ > 0) would exhibit both left-leg and right-leg habitual preference across the cohort. In other words, LTA_3D_ and *P* would be subject to the same (quasi-random) statistical distribution. If, however, a left-dominant lateral asymmetry (LTA_3D_ > 0) were associated with a habitual preference of the left leg (κ=1), and, therefore, right-leg PTA dominance (LTA_3D_ < 0) were linked to habitual right-leg preference (κ=−1), the product $P={\mathrm{LTA}}_{3D}\cdot \kappa \geq 0$ would be non-negative, thus clearly needing to show a different distribution of values compared to ${\mathrm{LTA}}_{3D}\frac{>}{<}0$.)

### 2.6 Statistical analysis

All statistical tests were performed using IBM SPSS Statistics (Version 29, Armonk, NY, USA). The significance level was set to *p* < *α* = 0.05 for all statistical tests. If not stated otherwise, all average values are given as mean ± standard deviation. The Shapiro–Wilk test was employed to check normality of the data, whilst the Mauchly test was utilised to determine the sphericity of the data.

For normally distributed data, the subsequent inferential statistical analysis was conducted using a three-way mixed-model analysis of variance (mixed ANOVA) with footwear condition (FWC) and running speed as within-subject factors, and sex as the between-subject factor. It was used to investigate the effects of FWC, running speed, sex, and their interactions on triaxial PTA (18). *Post-hoc* pairwise comparisons with Bonferroni correction were performed when mixed ANOVA yielded significant results. Partial eta squared (*η*_p_^2^) was calculated to estimate the effect size of the main effects, and interpreted according to Cohen (48, 49): Small effects for *η*_p_^2^ > 0.01, medium effects for *η*_p_^2^ > 0.06, large effects for *η*_p_^2^ > 0.14. In addition, the effect sizes of the *post-hoc* comparison were estimated by virtue of Cohen's *d* (*d*_Cohen_), with *d*_Cohen_ ≥ 0.2 representing a small effect, *d*_Cohen_ ≥ 0.5 a medium effect, and *d*_Cohen_ ≥ 0.8 a large effect (50, 51). For data that were not normally distributed, the aligned rank transform was conducted using the software ARTool (Version 2.2.2) (52). Subsequent analysis was conducted using a mixed ANOVA for each aligned rank condition separately (FWC, speed, FWC*speed). The objective was to investigate the effects of FWC, running speed, sex, and their interactions on RSh and LTA_3D_. For significant results, *post-hoc* pairwise comparisons were performed through Wilcoxon signed-rank test with Bonferroni correction. The estimation of effect sizes was then conducted by $r=|Z|/\sqrt{n}$, with *r* < 0.1 representing a negligible effect, 0.1 ≤ *r* < 0.3 a small effect, 0.3 ≤ *r* < 0.5 a medium effect, and *r* ≥ 0.5 a large effect. For the analysis of the influence of the dominant leg on LTA_3D_, a *χ*^2^ test was employed to test whether the distributions of LTA_3D_ and *P* were statistically equivalent, or, otherwise, if *P* was shifted towards non-negative values [cf. Equation (7)].

## 3 Results

The average speeds at which the ventilatory thresholds of the athletes were reached were *v*_VT1_ = 11.4 ± 1.1 km h^−1^ and *v*_VT2_ = 15.5 ± 1.6 km h^−1^, corresponding to average testing speeds of *v*_1_ = 10.3 ± 1.0 km h^−1^, *v*_2_ = 13.5 ± 1.3 km h^−1^ and *v*_3_ = 15.5 ± 1.6 km h^−1^. Group-level speeds for male and female athletes are displayed in Table 2.

**Table 2:** Running speeds at ventilatory thresholds 1 & 2 (*v*_VT1_ & *v*_VT2_) and speeds for the CPRS test (*v*_1_, *v*_2_ & *v*_3_) for all athletes as well as females only and males only.

Sub-cohort	Speed [km h^−1^]				
	*v*_VT1_ ± *σ*	*v*_VT2_ ± *σ*	*v*_1_ ± *σ*	*v*_2_ ± *σ*	*v*_3_ ± *σ*
All athletes	11.4 ± 1.1	15.5 ± 1.6	10.3 ± 1.0	13.5 ± 1.3	15.5 ± 1.6
Female	10.7 ± 0.7	14.2 ± 0.7	9.6 ± 0.6	12.5 ± 0.7	14.2 ± 0.7
Male	11.9 ± 1.0	16.4 ± 1.4	10.7 ± 0.9	14.2 ± 1.2	16.4 ± 1.4

*σ,* standard deviation, *v*_VT1_ & *v*_VT2,_ speed at ventilatory threshold 1 & 2, *v*_1_, *v*_2_ & *v*_3_, speed for the CPRS test, calculation described in methods.

### 3.1 Triaxial peak tibial acceleration

**Figure 2:**
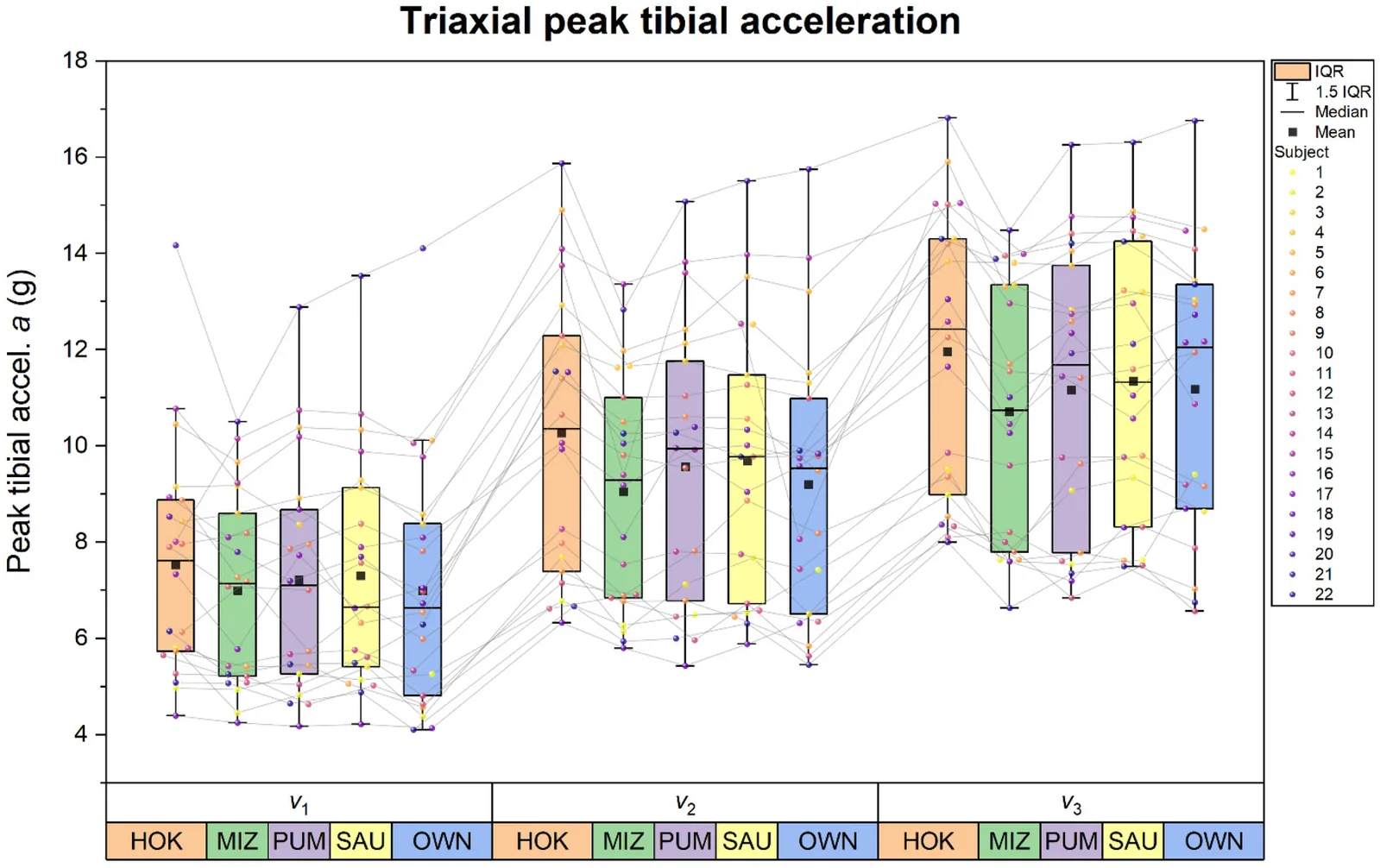
Distribution of triaxial peak tibial acceleration across five footwear conditions at the three running speeds *v*_1_, *v*_2_, and *v*_3_. Boxplots summarise the data (median, IQR, and 1.5 × IQR whiskers), while observations for individual participants are shown as dots connected by lines to illustrate within-subject changes across footwear conditions at each running speed. Footwear conditions: HOK (Hoka Rocket X2), MIZ (Mizuno Wave Rebellion Pro), PUM (Puma Fast-R Nitro Elite), SAU (Saucony Endorphin Pro 3) and OWN (own pair of non-carbon plate running shoes).

As illustrated in Figure 2, mean triaxial PTA increases with speed for the 5 FWCs. The mixed-model three-way ANOVA (Table 3) revealed large effects of FWC (*p* < 0.001, *η*_p_^2^ = 0.44) and running speed (*p* < 0.001, *η*_p_^2^ = 0.893) on PTA. It also showed significant interactions between FWC and sex (*p* = 0.008, *η*_p_^2^ = 0.157), and between speed and sex (*p* = 0.022, *η*_p_^2^ = 0.217). Given these significant interactions, the main effects for FWC and running speed were not analysed further.

**Table 3:** Results of mixed-model three-way ANOVA on PTA.

Effect	*p*		*η*_p_^2^	*df*	*F*	Sphericity correction
FWC	<0.001	*	0.440	4	15.721	Not required
FWC*Gender	0.008	*	0.157	4	3.715	Not required
Speed	<0.001	*	0.893	1.186	167.552	Greenhouse-Geisser
Speed*Gender	0.022	*	0.217	1.186	5.559	Greenhouse-Geisser
FWC*Speed	0.053		0.124	2.669	2.824	Greenhouse-Geisser
FWC*Speed*Gender	0.288		0.060	2.669	1.287	Greenhouse-Geisser

*, Statistically significant (*p* < 0.05), *η*_p_^2^, partial eta-squared, *df*, degrees of freedom, *F,* F-statistics.

Instead, when comparing both sexes, a significant difference was found between the group of male and female athletes for the FWC OWN (*p* = 0.015) with a small effect size (*d*_Cohen_ = 0.26). The CPRS FWC conditions, in turn, did not show significant differences between the sexes. When comparing the different FWC at the group level, the effect of FWC differed between males and females: In the females, significant differences in FWC were found for HOK vs. MIZ (*p* = 0.028), HOK vs. PUM (*p* = 0.008), HOK vs. OWN (*p* < 0.001), and SAU vs. OWN (*p* = 0.003). However, most effects were negligible (HOK vs. PUM: *d*_Cohen_ = 0.068; HOK vs. OWN: *d*_Cohen_ = 0.034; SAU vs. OWN: *d*_Cohen_ = 0.002). The only non-negligible, yet still small effect was found for HOK vs. MIZ (*d*_Cohen_ = 0.30). The male group showed slightly different results when comparing the different FWC. Significant differences were found for HOK vs. MIZ (*p* < 0.001), HOK vs. PUM (*p* = 0.003), HOK vs. SAU (*p* = 0.005), and MIZ vs. SAU (*p* = 0.047). Effect sizes were negligible for MIZ vs. SAU (*d*_Cohen_ = 0.08), small for HOK vs. PUM (*d*_Cohen_ = 0.42) and HOK vs. SAU (*d*_Cohen_ = 0.42), and medium for HOK vs. MIZ (*d*_Cohen_ = 0.53).

As for the interaction between speed and sex, a significant difference between males and females was found at *v*_3_ (*p* = 0.021), exhibiting a large effect size of *d*_Cohen_ = 1.09. When comparing the PTA at the group level for the different speeds, both the females’ and the males’ groups showed significant differences among all three speeds (*p* < 0.001 for *v*_1_ vs. *v*_2_, *v*_1_ vs. *v*_3_, and *v*_2_ vs. *v*_3_), with PTA generally increasing with running speed. The effect sizes in the females were large for *v*_1_ vs. *v*_2_ (*d*_Cohen_ = 2.86), *v*_1_ vs. *v*_3_ (*d*_Cohen_ = 3.66), and *v*_2_ vs. *v*_3_ (*d*_Cohen_ = 4.74). The males yielded similar result with large effect sizes for *v*_1_ vs. *v*_2_ (*d*_Cohen_ = 2.62), *v*_1_ vs. *v*_3_ (*d*_Cohen_ = 2.97), and *v*_2_ vs. *v*_3_ (*d*_Cohen_ = 2.52).

### 3.2 Residual shock

**Figure 3:**
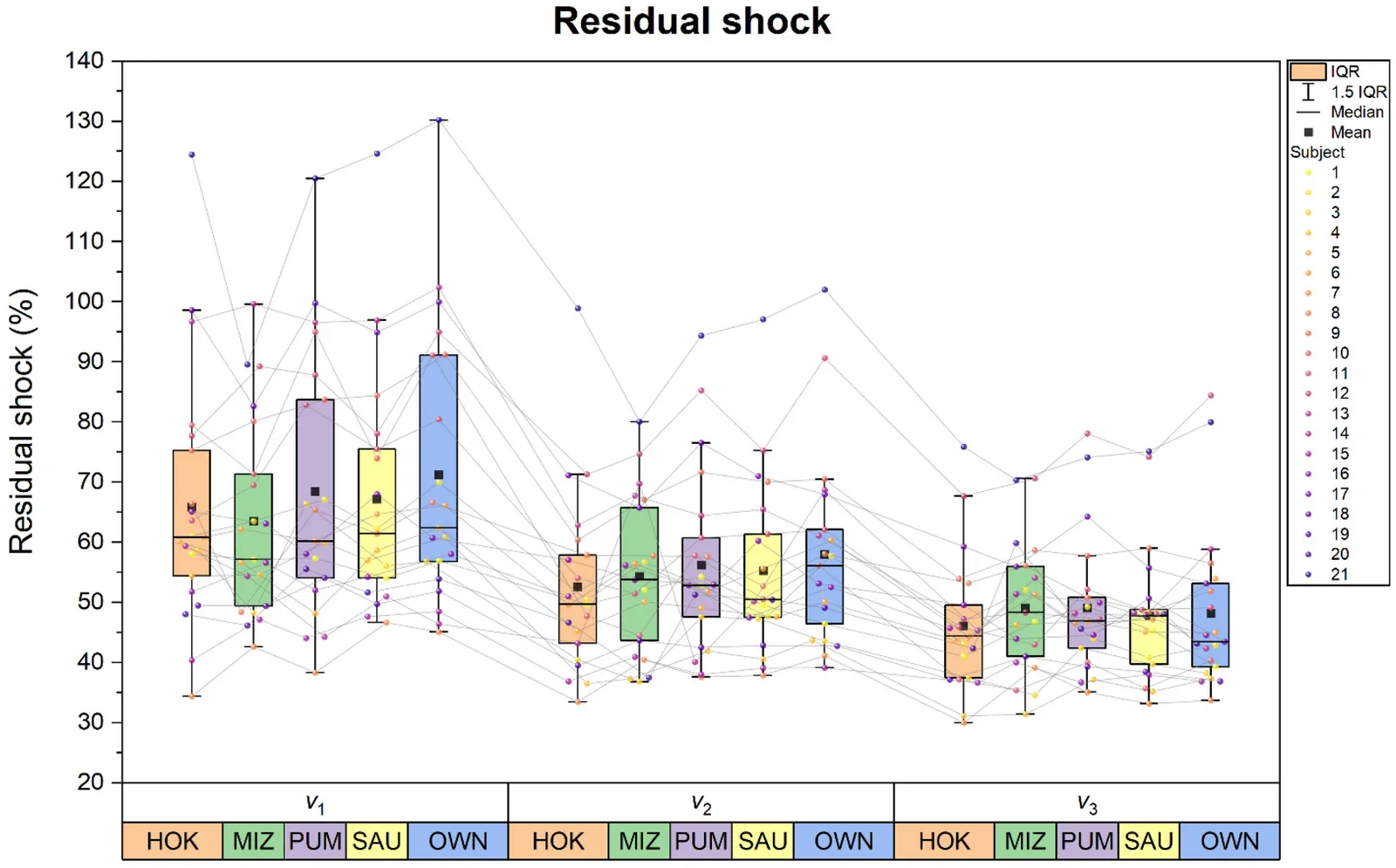
Distribution of residual shock across five footwear conditions at the three running speeds *v*_1_, *v*_2_, and *v*_3_. Boxplots summarise the data (median, IQR, and 1.5 × IQR whiskers), while observations for individual participants are shown as dots connected by lines to illustrate within-subject changes across footwear conditions at each running speed. Footwear conditions: HOK (Hoka Rocket X2), MIZ (Mizuno Wave Rebellion Pro), PUM (Puma Fast-R Nitro Elite), SAU (Saucony Endorphin Pro 3) and OWN (own pair of non-carbon plate running shoes).

For all FWC, residual shock decreased with increasing running speed from 63–71% at *v_1_* to 46–49% at *v_3_* (Figure 3). It was observed that a proportion of the athletes exhibited single values for *RSh* that exceeded one. This finding suggests that those athletes performed additional active movements of the body segments above the tibiae. Given that this was the case for all measurements of subject 22, it was deemed necessary to exclude that athlete from further analysis of *RSh*. As the Shapiro–Wilk test revealed that the data were not normally distributed (*p* = 0.001), the subsequent analysis of *RSh* was conducted using the using aligned rank transform and mixed ANOVA. The mixed ANOVA yielded significant results for FWC (*p* = 0.021, *η*_p_^2^ = 0.140) and speed (*p* < 0.001, *η*_p_^2^ = 0.704) with the effect sizes being large (Table 4). In addition, it revealed significant interactions between FWC and running speed (*p* = 0.005, *η*_p_^2^ = 0.176). Owing to this significant interaction, the main effects for FWC and running speed were not analysed further.

**Table 4:** Results of mixed-model three-way ANOVA on *RSh* after aligned rank transform.

Effect	*p*		*η*_p_^2^	*df*	*F*	Sphericity correction
FWC	0.021	*	0.140	4	3.085	Not required
FWC*Gender	0.542		0.039	4	0.780	Not required
Speed	< 0.001	*	0.704	1.307	45.228	Greenhouse-Geisser
Speed*Gender	0.472		0.033	1.307	0.639	Greenhouse-Geisser
FWC*Speed	0.005	*	0.176	3.947	4.055	Greenhouse-Geisser
FWC*Speed*Gender	0.067		0.108	3.947	2.307	Greenhouse-Geisser

*, Statistically significant (*p* < 0.05), *η*_p_^2^, partial eta-squared, *df*, degrees of freedom, *F*, F statistic.

To analyse the interaction between FWC and speed, the five FWCs were compared at the three different speeds (Table 5). Statistically significant results were found at *v_1_* for HOK vs. OWN (*p* = 0.033, *r* = 0.64), MIZ vs. OWN (*p* = 0.008, *r* = 0.73), and SAU vs. OWN (*p* = 0.041, *r* = 0.63). For *v_2_*, statistically significant results were found for HOK vs. PUM (*p* = 0.033, *r* = 0.64) and HOK vs. OWN (*p* = 0.037, *r* = 0.63). Finally, at *v_3_*, one statistically significant result was found for the interaction between HOK and PUM (*p* = 0.024, *r* = 0.66). All significant results showed large effect sizes.

**Table 5:** *Post-hoc* Wilcoxon signed-rank pairwise comparison of the five FWCs at the three different speeds for *RSh*.

FWC_*i*_ vs. FWC_*j*_		*v*	*p*		*r*	Effect size
HOK	MIZ	*v*_1_	1.000		0.09	negligible
		*v_2_*	1.000		0.29	small
		*v*_3_	0.298		0.47	medium
HOK	PUM	*v*_1_	1.000		0.33	medium
		*v_2_*	0.033	*	0.64	large
		*v*_3_	0.024	*	0.66	large
HOK	SAU	*v*_1_	1.000		0.19	small
		*v_2_*	0.087		0.57	large
		*v*_3_	0.208		0.50	large
HOK	OWN	*v*_1_	0.033	*	0.64	large
		*v_2_*	0.037	*	0.63	large
		*v*_3_	1.000		0.30	small
MIZ	PUM	*v*_1_	1.000		0.35	medium
		*v_2_*	1.000		0.25	small
		*v*_3_	1.000		0.00	negligible
MIZ	SAU	*v*_1_	0.386		0.45	medium
		*v_2_*	1.000		0.13	small
		*v*_3_	1.000		0.21	small
MIZ	OWN	*v*_1_	0.008	*	0.73	large
		*v_2_*	0.630		0.41	medium
		*v*_3_	1.000		0.13	small
PUM	SAU	*v*_1_	1.000		0.12	small
		*v_2_*	1.000		0.25	small
		*v*_3_	0.420		0.44	medium
PUM	OWN	*v*_1_	0.273		0.48	medium
		*v_2_*	1.000		0.16	small
		*v*_3_	1.000		0.26	small
SAU	OWN	*v*_1_	0.041	*	0.63	large
		*v_2_*	0.735		0.39	medium
		*v*_3_	1.000		0.01	negligible

*, Statistically significant (*p* < 0.05), *r,* effect size of the Wilcoxon signed-rank test. Footwear conditions: HOK, Hoka Rocket X2; MIZ, Mizuno Wave Rebellion Pro; PUM, Puma Fast-R Nitro Elite; SAU, Saucony Endorphin Pro 3; and OWN, own pair of non-carbon plate running shoes.

### 3.3 Triaxial tibial asymmetry

#### 3.3.1 Mean cohort results

**Figure 4:**
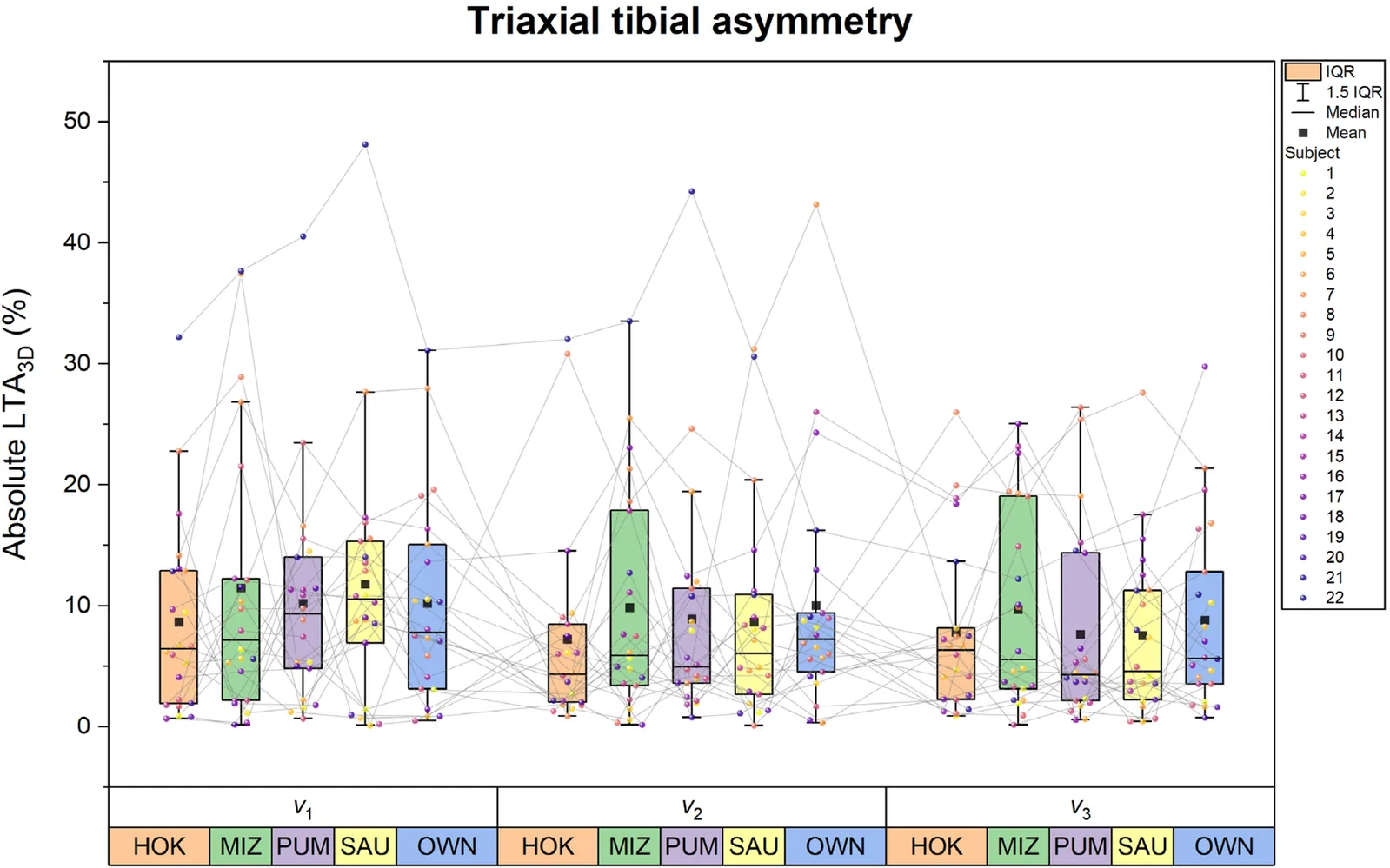
Distribution of LTA_3D_ across five footwear conditions at the three running speeds *v*_1_, *v*_2_, and *v*_3_. Boxplots summarise the data (median, IQR, and 1.5 × IQR whiskers), while observations for individual participants are shown as dots connected by lines to illustrate within-subject changes across footwear conditions at each running speed. Footwear conditions: HOK (Hoka Rocket X2), MIZ (Mizuno Wave Rebellion Pro), PUM (Puma Fast-R Nitro Elite), SAU (Saucony Endorphin Pro 3) and OWN (own pair of non-carbon plate running shoes).

As the Shapiro–Wilk test revealed non-normally distributed data (*p* < 0.001) for LTA_3D_, the subsequent analysis for LTA_3D_ was conducted using aligned rank transform and mixed ANOVA. The mixed ANOVA and the subsequent pairwise comparison showed no statistically significant results (Table 6) between the different FWC and speeds (*p* = 0.240). Figure 4 depicts the absolute value of LTA_3D_ for the whole cohort across all FWC and speeds, ranging from minimal mean values of 7.21% for HOK at *v*_2_ to maximal mean values of 11.76% for SAU at *v*_1_.

**Table 6:** Results of mixed-model three-way ANOVA on LTA_3D_ after aligned rank transform.

Effect	*p*	*η*_p_^2^	*df*	*F*	Sphericity correction
FWC	0.496	0.041	4	0.852	Not required
FWC*Gender	0.367	0.041	4	0.076	Not required
Speed	0.240	0.069	2	1.478	Not required
Speed*Gender	0.798	0.011	2	0.227	Not required
FWC*Speed	0.771	0.029	8	0.607	Not required
FWC*Speed*Gender	0.802	0.028	8	0.569	Not required

*, Statistically significant (*p* < 0.05), *η*_p_^2^, partial eta-squared, *df,* degrees of freedom, *F,* F-statistics.

#### 3.3.2 Individual results

**Figure 5:**
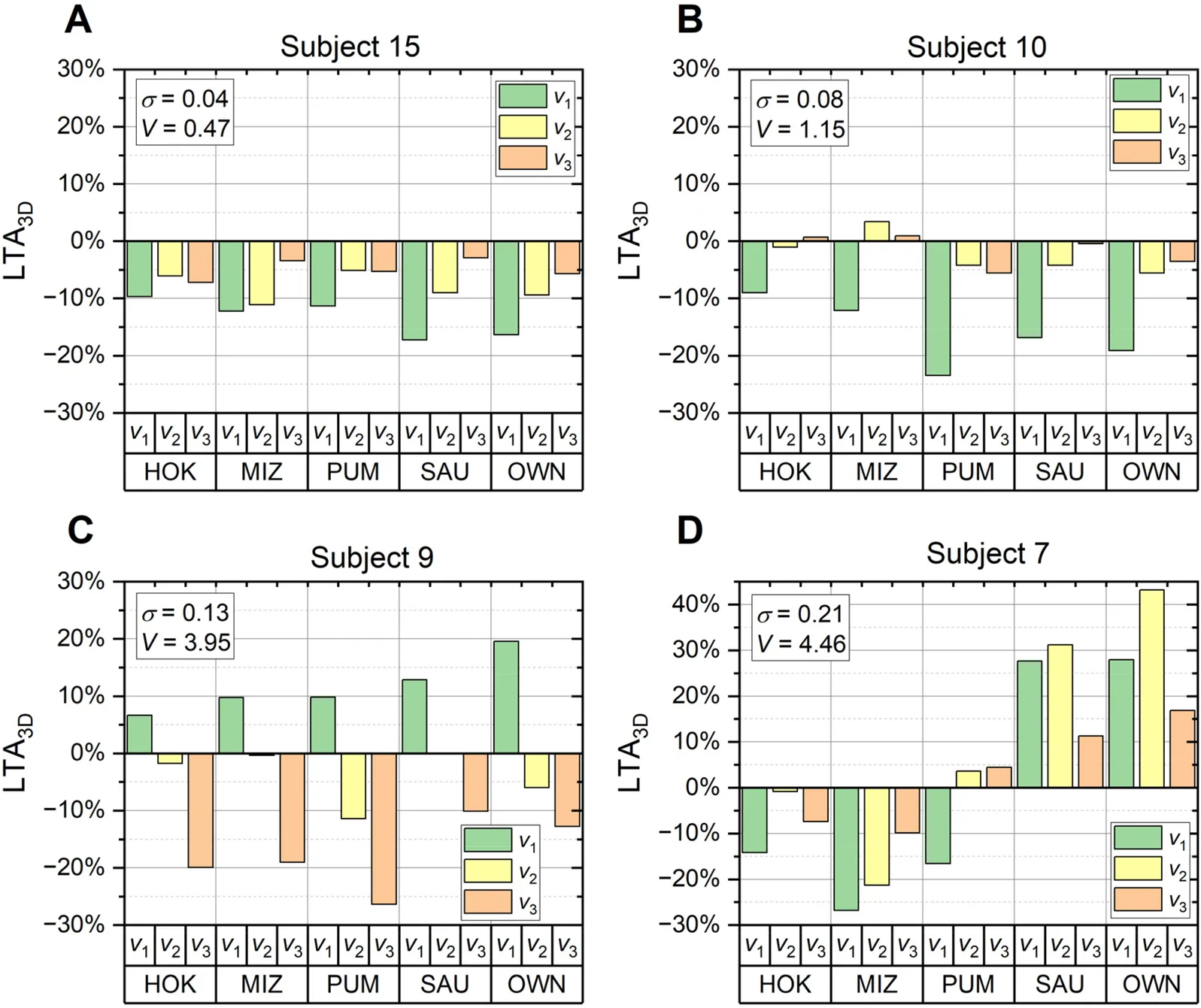
**(A–D)** Examples of LTA_3D_ (triaxial lateral tibial asymmetry) of 4 out of 22 subjects at the three running speeds *v*_1_, *v*_2_ and *v*_3_ for the five footwear conditions HOK (Hoka Rocket X2), MIZ (Mizuno Wave Rebellion Pro), PUM (Puma Fast-R Nitro Elite), SAU (Saucony Endorphin Pro 3) and OWN (own pair of non-carbon plate training shoes). *σ* = standard deviation, *V* = coefficient of variation.

On the individual level, the analysis of the LTA_3D_ reveals that the outcome differs substantially between subjects. Figures 5A–D illustrates this high inter-individual variance of FWC effects based on four exemplary athletes: Subject 15, for instance, demonstrates LTA_3D_ at a stable level of 8.8 ± 4.3% across all FWCs and speeds. In contrast, Subject 10 exhibits a decline in LTA_3D_ with increasing running speed from 14.6 ± 8.3% at *v*_1_ to 2.3 ± 2.2% at *v*_3_. Which is virtually independent of FWC. As depicted in the panel of Subject 9, that subject showed an increase in LTA_3D_ with higher running speeds. In addition, there is a concomitant change of the sign of the asymmetry from one leg to the other as speed increases. In contrast to those examples, Subject 7 does not seem to follow a systematic trend but rather demonstrates varying degrees of LTA_3D_ across the FWCs and speeds.

#### 3.3.3 Influence of the dominant leg on Lta_3d_

The *χ*^2^ test to illicit any potential link between lateral asymmetry and leg dominance (Table 7) revealed no statistically significant difference (*p* = 0.240) between the distributions of LTA_3D_ and *P*, implying that habitual leg dominance had *no* effect on kinematic leg asymmetry or vice versa [see Section 2.5, particularly explanation following Equation (7)].

**Table 7:** Self-reported leg dominance of the participants.

Criterion	Left leg dominant	Right leg dominant	Indifferent leg dominance
Jumping leg	11	11	0
Stance leg	22	0	0

## 4 Discussion

The aim of this study was to determine the effects of CPRS compared to non-CPRS on the biomechanical load metrics PTA, residual shock and lateral asymmetry index.

Our results show that PTA was increased with running speed in both sexes, an effect that is known from non-CPRS (18). However, at the highest running speed *v*_3_, statistically significant differences in PTA were observed between the sexes. This phenomenon may be attributable to the divergent running speed levels exhibited by the two gender groups, with the male subjects running approximately 15% faster than the females at *v*_3_. At this point, it may be argued that the sex-specific findings of this study should be treated cautiously because the initial power analysis had been conducted for a combined group of both sexes (*n* ≥ 21), whereas a sex-specific analysis of biomechanical metrics may require a higher number of runners per sex than the actual 13 men and 9 women participating in this study. However, because the type I error associated with the pertinent statistical analysis is still controlled by *α* < 0.05, the significant result for sex-specific differences observed in this study remains statistically valid and sound, even though the corresponding type II error may have exceeded the initially defined value *β* = 0.2 (favouring false-negative results instead of false-positives).

Moreover, the observed trend of an increase in PTA with increasing speed on a group level is consistent across both groups. When comparing the FWCs for each sex separately, HOK showed significant differences from the other FWCs, with HOK having higher PTA values. Contrary to the assumption that more cushioning may lead to lower PTA, it was shown in previous research that more cushioning actually results in higher PTA, at least in non-CPRS (12). This inverse relationship between the level of cushioning and PTA amplitudes is likely to be one reason why HOK produces higher PTA values in the subjects. Further research is needed to validate this hypothesis. It can be concluded that CPRS do not substantially change the amplitudes of PTA in comparison to non-CPRS. Possibly, the comparable PTA may suggest, on the individual level, that also internal tibial loads may be similar. However, this remains a debatable hypothesis in view of the fact that both intra- and inter-individual correlations between PTA and actual bone loading have been shown to be only weekly correlated (19, 20).

The residual shock exhibited a decreasing trend with increasing running speed. This indicates that, at higher speeds, there is a reduction in the acceleration of the tibia that is transmitted to the sacrum. The residual shock levels exhibited a range from about 63% to 71% at *v*_1_, and 46% to 49% at *v*_3_. This finding aligns with the results reported in the extant literature on non-CPRS (18, 45, 53), suggesting a comparable response of CPRS in this regard. However, upon examination of the absolute values for residual shock, it was found that the mean proportion of residual shock that is measurable at the sacrum of ≈57% is larger than in previous studies, where ≈32% and ≈48% were reported (18, 44). This is most probably attributable to the difference in athletic populations participating in this study: In contrast to previous studies (18, 44, 45), this study recruited amateur runners of both sexes instead of elite runners or male runners only. Another potential explanation for the higher values of residual shock observed in this study could be attributed to the softer nature of the treadmill used in this investigation, as compared to the more rigid treadmills employed in previous studies (18, 44, 45). At fixed running speeds significant differences in residual shock were observed between some of the FWCs. The residual shock appeared to be lower for CPRS than for OWN in a few cases, namely at *v_1_* for HOK (≈66%), MIZ (≈63%) and SAU (≈67%) as compared to OWN (≈71%), and at *v_2_* for HOK (≈53%) as compared to OWN (≈58%). However, the substantial structural heterogeneity in the OWN condition (see below) may have contributed to this statistical outcome. Moreover, HOK showed statistically significantly lower *RSh* values than PUM at *v_2_* (≈53% for HOK, and ≈56% for PUM) and *v_3_* (≈46% for HOK, and ≈49% for PUM). As HOK frequently differed from other footwear models in our study, the observed effects may partly be attributed to the specific cushioning properties of the HOK shoe, which has been reported to be more compliant than other CPRS shoes. This increased cushioning has been shown to result in higher PTA and consequently reduced residual shock (12). This may likewise be applicable in the context of the observation that non-CPRS show elevated levels of residual shocks, as the cushioning appears to be lower in comparison with that of CPRS. Therefore, future research should explicitly investigate the influence of different foams on residual shock in CPRS.

As to the third key result of this study, the asymmetry index, CPRS did not affect the degree of lateral asymmetry in PTA during running. Median asymmetry indices ranged from 7.5 to 11.8% across all speeds, with these values being described as physiological in previous studies (21, 33). Hence, higher cushioning and energy recoil observed in the CPRS of this study did not appear to increase existing asymmetries, a finding that is consistent with the notion that these asymmetries manifest to a comparable extent in both legs. Therefore, it can be concluded that the extent to which asymmetries occur in runners is attributable to an intrinsic individual factor (23). Furthermore, no statistical association between the dominant leg and the side of lateral asymmetry was observed, meaning that the side of the dominant leg (i.e., left or right) was not linked to the side of lateral asymmetry. This result is in line with a recent finding that the dominant leg in terms of PTA or ground reaction forces, respectively, is not associated with the side of a specific overuse injuries in runners (24).

An analysis of the individual results reveals a variety of peculiarities of runners, each responding differently to CPRS. Some of the runners demonstrated a decline in triaxial tibial asymmetry with increasing running speeds. This finding is consistent with the observation in elite athletes reported previously (23). It is argued that such elite runners improve their efficiency in running at higher speeds and exhibit an optimised locomotion pattern. By contrast, the present study also revealed that some runners demonstrate an increasing degree of asymmetry as their running speed increases. This effect may be due to a lower training level of several of the runners participating in this study. It is reasonable to hypothesise that some less trained amateur runners may experience a loss of stability with increasing degree of exhaustion, which is likely to result in a deviation from their optimally balanced running gait. Furthermore, some of the runners in this study demonstrated a relatively consistent degree of LTA_3D_ across all FWCs and running speeds. This finding may indicate that neither exhaustion nor enhancement in running technique exert an influence on their individual asymmetry. Finally, other runners exhibited no clear pattern, but rather different reactions to each FWC, resulting in varying degrees of LTA_3D_ in each FWC.

Altogether, the different responses of the athletes to CPRS show that CPRS may either substantially alter the individual degree of lateral asymmetry in running gait compared to their preferred non-CPRS, both in terms of increase (see, e.g., athletes 11, 20 for MIZ in Figure S1) or decrease (see, e.g., athletes 7, 13 for HOK in Figure S1), or may leave it as is (see athletes 12, 15 for SAU in Figure S1). Therefore, we recommend to first individually test if a specific CPRS substantially changes LTA_3D_, before using it in training or competition. By virtue of a variety of suitable and affordable IMUs available on the market today, this does not seem to be an unrealistic option any longer.

Some limitations of this study must be noted. First, due to restrictions in budget and time, this study could only test 4 different CPRS, and only one test day per athlete could be performed with the CPRS. Second, the effect of fatigue could not be accounted for as each FWC was tested in a random order, without a randomisation of the speeds. The randomisation of the order in which the shoes were tested was deemed to be the most appropriate approach in order to avoid any potential bias between the FWC tested. Furthermore, because of the randomisation of the shoes, each FWC was used in each position (1–5), resulting in statistically similar fatigue effects for each FWC. To investigate the influence of fatigue on, e.g., the asymmetry index when wearing CPRS in future studies, incorporating longer intervals and a larger number of test days seem advisable. Moreover, no stand-alone testing of the pure mechanical properties of the shoes could be conducted (e. g., measuring midsole hardness, differences in carbon plate geometry etc.) because our laboratory does not allow for such procedures. Finally, it is also important to note that the shoe condition OWN was individual for each athlete, thereby representing a variety of different footwear metrics such as stack height, stiffness and drop. In particular, the mass of individual shoes in the OWN condition differed by a maximum of 147 g (range 188–335 g; the latter figure representing an outlier), whereas the mass of individual shoes in the four CPRS conditions differed by only 45 g (range 186–231 g), representing only a third of the difference observed in OWN. Therefore, this substantial structural heterogeneity in OWN may have obscured any genuine differences between CPRS and simple footwear. This limits the comparison of the CPRS FWCs as a whole to OWN, but is required to enable a comparison of the individual asymmetry index among different FWCs, as only the individual own FWC can safely be considered a valid reference for individual asymmetry in PTA. To this end, Supplementary Figure 1 compares the LTA_3D_ asymmetry metric at the individual level for all subjects separately.

## 5 Conclusion

This study provides insight into the alterations in biomechanical parameters (PTA, residual shock and lateral asymmetry index) that individuals can expect when wearing CPRS. At cohort level, PTA differed between footwear models and increased with running speed. In contrast, residual shock showed limited footwear-related differences and generally decreased with increasing speed. No group-level effects of FWC or speed were observed for the lateral asymmetry index. However, as the non-carbon plate condition OWN was highly heterogeneous by design, the comparability between CPRS and simple footwear technology may be limited. Notwithstanding, sex-specific differences and inter-individual variability were evident across the measured parameters. These findings highlight that responses to CPRS are highly individual. From a practical perspective, athletes and practitioners may therefore benefit from evaluating different CPRS models on an individual basis when aiming to identify the most suitable footwear for a given runner and use case.

## Data Availability

The raw data supporting the conclusions of this article will be made available by the authors, without undue reservation.
