# Supplementary material for: Effect of various carbon plate running shoes on peak tibial accelerations, lateral asymmetry and residual shock in running
Source: Front Sports Act Living. 2026 Sep 4;8:1908480. doi: 10.3389/fspor.2026.1908480 (PMC13585708; doi:10.3389/fspor.2026.1908480)
Supplement: Supplementary file 1 [file Datasheet1.docx]

Supplementary Material

Supplementary Figure 1. LTA3D of all 22 subjects at the three running speeds v1, v2 and v3 for the five footwear conditions HOK (Hoka Rocket X2), MIZ (Mizuno Wave Rebellion Pro), PUM (Puma Fast-R Nitro Elite), SAU (Saucony Endorphin Pro 3) and OWN (own pair of non-carbon plate training shoes).

Supplementary Table 1. Basic structural properties for all OWN models used.

|  | Adidas | | Brooks | | Hoka | New Balance | Nike | | | | | Saucony | Under Armour |
| --- | --- | --- | --- | --- | --- | --- | --- | --- | --- | --- | --- | --- | --- |
|  | Adizero Adios 7 | Solar Boost 3 | Glycerin 20 | Levitate 5 | Mach 5 | Fresh Foam X More v4 | Pegasus 37 | Pegasus 38 | Pegasus 39 | Pegasus Turbo 2 | React Infinity Run (1^st^ Generation) | Endorphin Speed (1^st^ Generation) | HOVR Machina (1^st^ Generation) |
| Release year^1^ | 2022 | 2021 | 2022 | 2021 | 2022 | 2022 | 2020 | 2021 | 2022 | 2019 | 2020 | 2020 | 2020 |
| Mass (men)* | 240 g (US 11) | 335 g (US 11.5) |  | 278 g (US 7) | 240 - 252 g (US 10.5 - 11.5) |  | 265 g (US 7.5) | 265 - 270 g (US 8.5 - 9) |  | 220 g (US 10) |  | 221 g (US 9.5) | 286 g (US 9) |
| Mass (women)* |  |  | 226 g (US 9) |  | 188 - 219 g (US 7.5 - 10) | 238 (US 8) |  |  | 252 g (US 9) | 214 g (US 10.5) | 229 g (US 8) |  |  |
| Heel drop^1^ | 8 mm | 10 mm | 10 mm | 8 mm | 5 mm | 4 mm | 10 mm | 10 mm | 10 mm | 10 mm | 9 mm | 8 mm | 8 mm |
| Reference | S1 | S2 | S3 | S4 | S5 | S6 | S7 | S8 | S9 | S10 | S11 | S12 | S13 |

Abbreviations: * measured in the laboratory for the sizes indicated; ^1^ data taken from the references in the last row of the table above.

# 1 References

S1	https://www.runnea.de/laufschuhe/adidas/adizero-adios-7/1014717/

S2	https://pushing-limits.de/test/adidas-solarboost-3-im-test/

S3	https://www.laufschuhkauf.de/2022/10/laufschuhtest-brooks-glycerin-20/

S4	https://www.laufschuhkauf.de/2022/01/laufschuhtest-brooks-levitate-5/

S5	https://www.runningxpert.com/de/inspiration/hoka-mach-5

S6	https://21run.com/blog/2023/03/new-balance-fresh-foam-x-more-v4/?srsltid=AfmBOoqMWreln5HgvaTsM3p32KpTU_sFH_pkfRDfb8eDnsk13XBsoSHk

S7	https://pushing-limits.de/test/nike-pegasus-37-im-test/

S8	https://www.running-culture.de/nike-pegasus-38-test/

S9	https://shop4runners.com/blog/post/pegasus-39-im-test

S10	https://www.solereview.com/nike-zoom-pegasus-turbo-2-review/

S11	https://pushing-limits.de/test/nike-react-infinity-run-flyknit-im-test/

S12	https://www.laufschuhkauf.de/2021/01/laufschuhtest-saucony-endorphin-speed/

S13	https://www.runningxpert.com/de/inspiration/hoka-mach-5
